# Supplementary material for: ER Stress Response and Induction of Apoptosis in Malignant Pleural Mesothelioma: The Achilles Heel Targeted by the Anticancer Ruthenium Drug BOLD-100
Source: Cancers (Basel). 2022 Aug 26;14(17):4126. doi: 10.3390/cancers14174126 (PMC9454852; doi:10.3390/cancers14174126)
Supplement: Supplementary file 1 [file cancers-14-04126-s001.zip › cancers-1804415-supplementary.pdf]

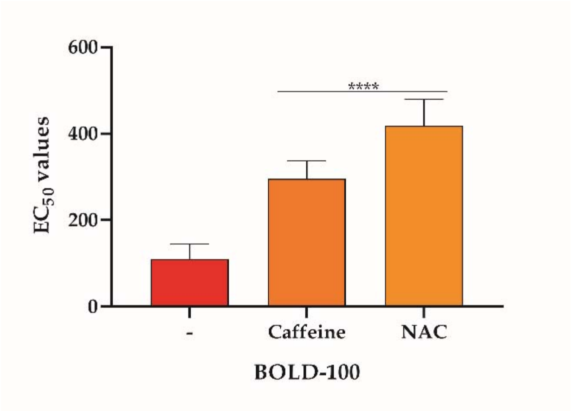

Supplementary Figure S1. Pretreatment with caffeine or NAC reduced BOLD-100 cytotoxicity in REN cells. Cell viability was evaluated in terms of EC_{50} at 24 h. Each value comes from the results of three independent experiments. Asterisks on bars indicate statistical differences (**** $p < 0.0001$, One-way ANOVA follow by Dunnett post-test).

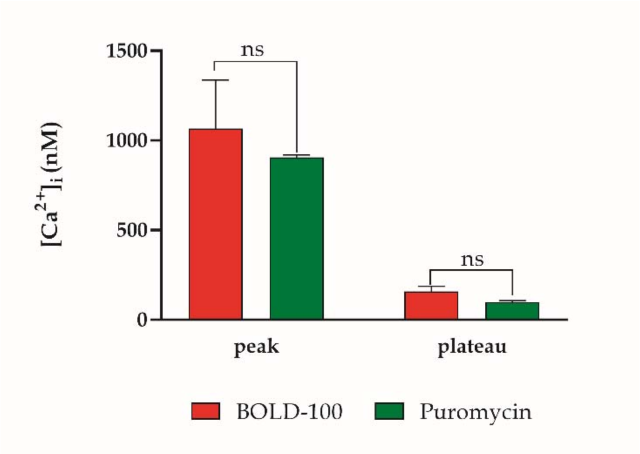

Supplementary Figure S2. Comparison of $[Ca^{2+}]_i$ variation recorded at 5-s intervals in REN cells after BOLD-treatment or 2 µM puromycin. Data are means± s.e.m. of Ca^{2+} peak response recorded in different cells. Number of cells: 40 cells from 3 exp for each condition. Statistics indicate differences at peak or plateau level for each condition (ns not significant, Two-way ANOVA follow by Bonferroni correction).
